# Hunterian Society

**Published:** 1830-04-01

**Authors:** 


### HUNTERIAN SOCIETY.

In the annual report of this Society's meetings, we find an animated appeal to the profession on the advantages of medical associations of this kind; and we wish the appeal may produce the desired effect of stimulating the members of our profession to more union and cordiality than have hitherto prevailed. "By free conference," say they, "and discussion among men varying in their talents and bias, difficult points are most successfully elucidated. The subject is represented in all its forms and bearings: one individual supplies light from past ages—another from the purer sources of the present—and all combine the testimony of their own observations and experience. In this way, occurrences related to one another, but having appearances of dissimilarity—and others seemingly analogous, but widely different—are seen in their true characters; so that obscure and mistaken facts, of high importance, which otherwise would have been lost, are made subservient to the knowledge and treatment of disease."

A prize is offered by the Society for the best paper on tubercular diseases.

---

